# Strategy to track double-J stents placed during COVID-19 using smartphone-based stent tracker application to prevent forgotten double-J stent in a high-volume centre: a smart solution

**DOI:** 10.1186/s12301-021-00212-3

**Published:** 2021-07-27

**Authors:** Abhirudra Mulay, Rohit Kapoor, Sonu Sharma, Shashikant Asabe, Hareesh Belagali, Siddharth Singh, Vikram Satav, Vilas Sabale

**Affiliations:** Department of Urology, Dr. D. Y. Patil Medical College and Research Centre, Renal Transplantation and Robotic Surgery, Pimpri-Chinchwad, 411018 India

**Keywords:** Forgotten DJ stent, Stent tracker application, COVID-19, Smartphones

## Abstract

**Background:**

Forgotten or retained (double-J) DJ stents may lead to several complications. Management of retained DJ stents poses a challenge for urologists not just surgically but also medicolegally and adds to the economic burden of the patient. Difficulty in follow-up for patients due to the contagious nature of COVID-19 and several restrictions posed in the form of lockdown. Smartphones today have become an integral part of our daily lives providing a convenient and reliable platform for data storage and access.

**Methods:**

All patients requiring placement of DJ stents and agreeing to enrol in the study were registered on the application over the physicians smartphone. SMSs regarding dates for removal of stent and follow-up with the literature regarding stent care were sent to the patients in their regional language.

**Results:**

A total of 100 patients were stented during this period of 3 months. Mean age was 42.61 years with three patients of paediatric age group. Mean duration of stent was 6 weeks. All patients received periodic messages (average 3) regarding follow-up and date for stent removal. 3%(*n* = 3) patients were delayed for follow-up; 2% (*n* = 2) patients lost to follow-up, compared to a 9% to 10% loss to follow-up in patients followed up only on paper discharges in our department previously.

**Conclusion:**

It significantly reduced the number of physical visits of the patient to the hospital and provided a more streamlined tracking of the indwelling stents for the user; patient compliance was found to be almost 98%; being cloud based (android/iOS), it was easily accessible to the user; and with the option of sharing the account details, the patient record could be accessed by several residents from their individual devices, which significantly reduced loss to follow-up rates from 9 to 2%.

## Background

Ureteral double-J (DJ) stenting is one of the most common procedures done in daily urological practice both as a day care procedure and under elective anaesthesia. DJ stent is an indispensable first-line device, in the armamentarium of a urologist. The placement of double-J (DJ) ureteral stent has several indications, such as in the treatment of urolithiasis, to relieve benign or malignant obstruction, to promote ureteral healing, and in the management of urinary leak [1]. DJ stents have mostly been placed as a modality of treatment to temporarily bypass an obstructed urinary system requiring timely removal or periodic changing depending upon the indication for insertion and the material of stent used.

Forgotten ureteral stent (FUS) carries a substantial risk of increasing morbidity in the patient. It has the potential to turn a double-J (DJ) stent placed for therapeutic purposes into a source of pathology. Management of retained DJ stents poses a challenge for urologists not just surgically but also medicolegally and adds to the economic burden of the patient. Forgotten or retained DJ stents may lead to complications ranging from stent migration, occlusion, breakage, encrustation [2] to being a source of urinary tract infections and sepsis and may even lead to more severe complications such as renal failure and mortality [3]. In the light of the recent COVID-19 pandemic, elective urological cases both at our institute and globally had been halted. Due to the contagious nature of the disease and several restrictions posed in the form of lockdown, it would be difficult for a patient to regularly follow-up post-placement of stent over the regular paper discharge or OPD card, which themselves might pose as a source of fomite infection.

Smartphones today have become an integral part of our daily lives providing a convenient and reliable platform for data storage and access. Stent Tracker application was developed in association with INTAS pharmaceuticals, with the aim to avoid forgotten or retained ureteric DJ stents and to provide an organized and convenient platform for tracking of these stents placed during the lockdown period.

## Aim of the study

The aim of this study was to avoid forgotten or retained ureteric DJ stents and to provide an organized, convenient and accessible online platform for tracking of these stents placed during the lockdown period and as a potential for future use.

## Method

We analysed all double-J ureteral stents registered in the Stent Tracker® between March 2020 and May 2020 at our institute.

All patients enrolled in the study were explained in detail about the working of the application, and informed and written consents were taken before enrolling the patients. All patients requiring placement of DJ stents and agreeing to enrol in the study were included.

The exclusion criteria included unwillingness of the patient to enrol.

**Fig. 1:**
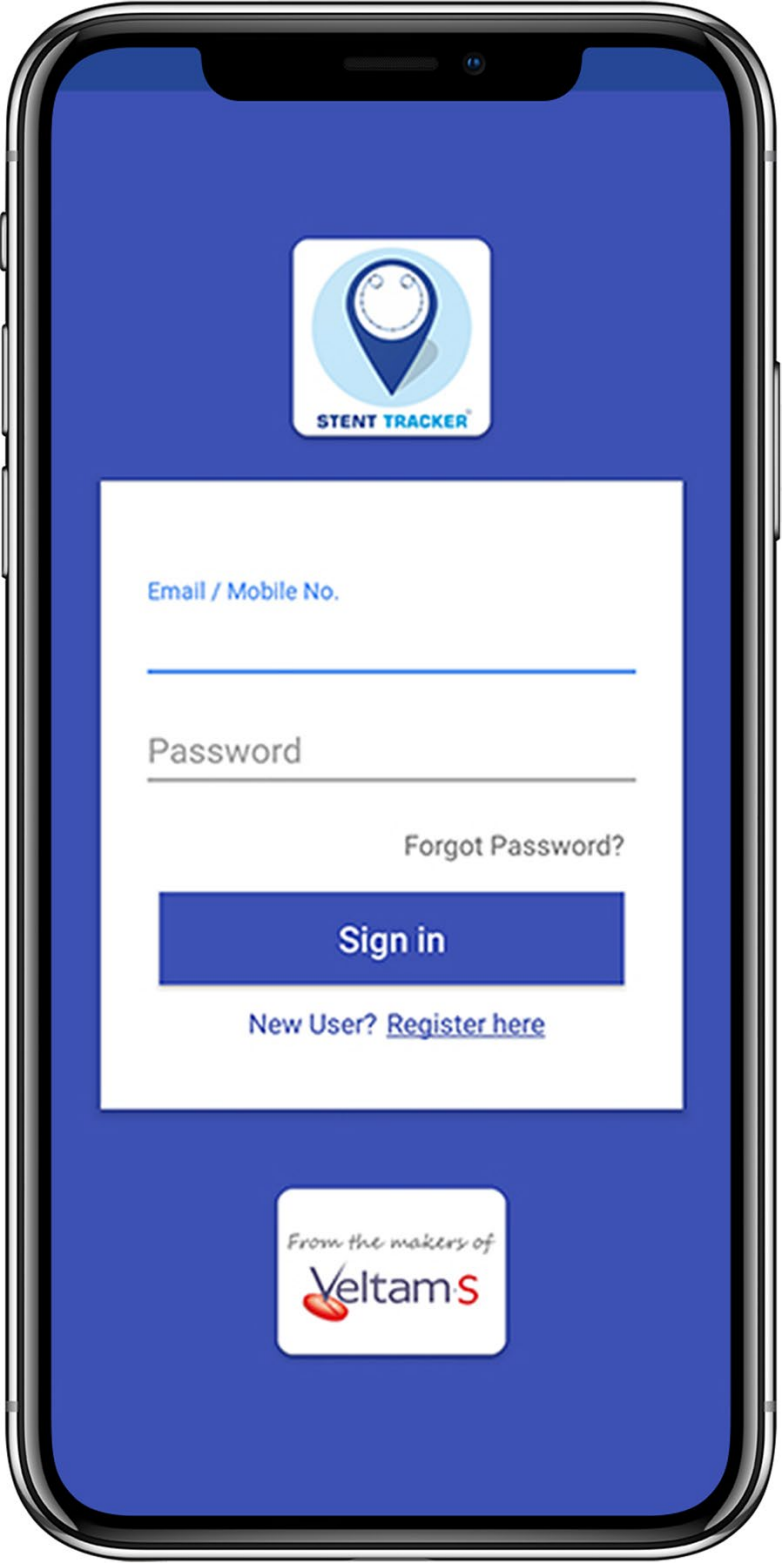
Login page of the application

**Fig. 2:**
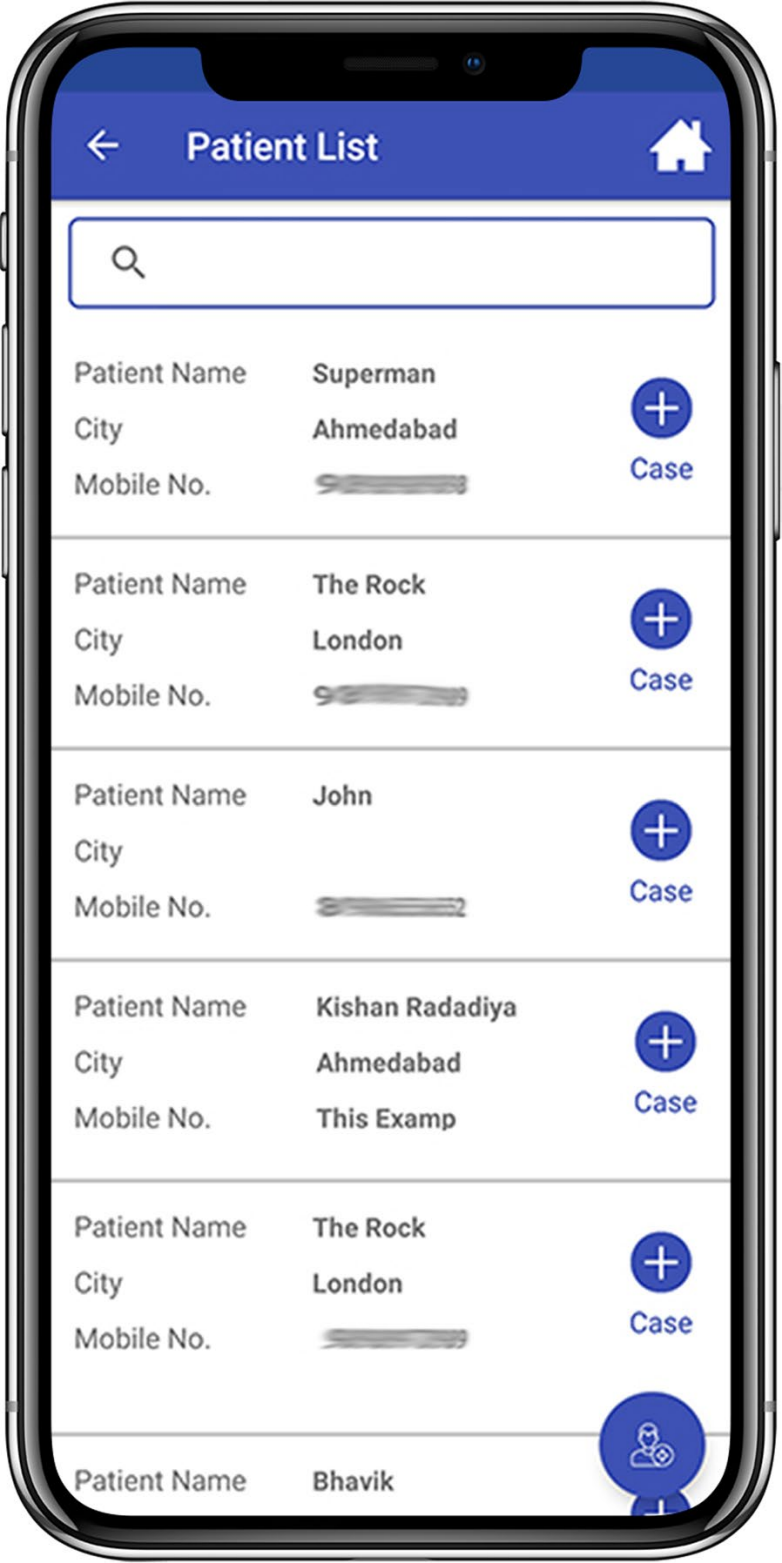
Patient list

Stent Tracker ® is designed by Intas Pharmaceuticals Ltd to reduce the incidence of forgotten ureteral stents. Once the stent had been inserted, user needs to add ‘stent episode’ in Stent Tracker ®. User needs to add patient details and create a case upon stent insertion. On successful registration of the patient, a message is sent to user as well as patient via SMS (Figs. 1, 2).

**Fig. 3:**
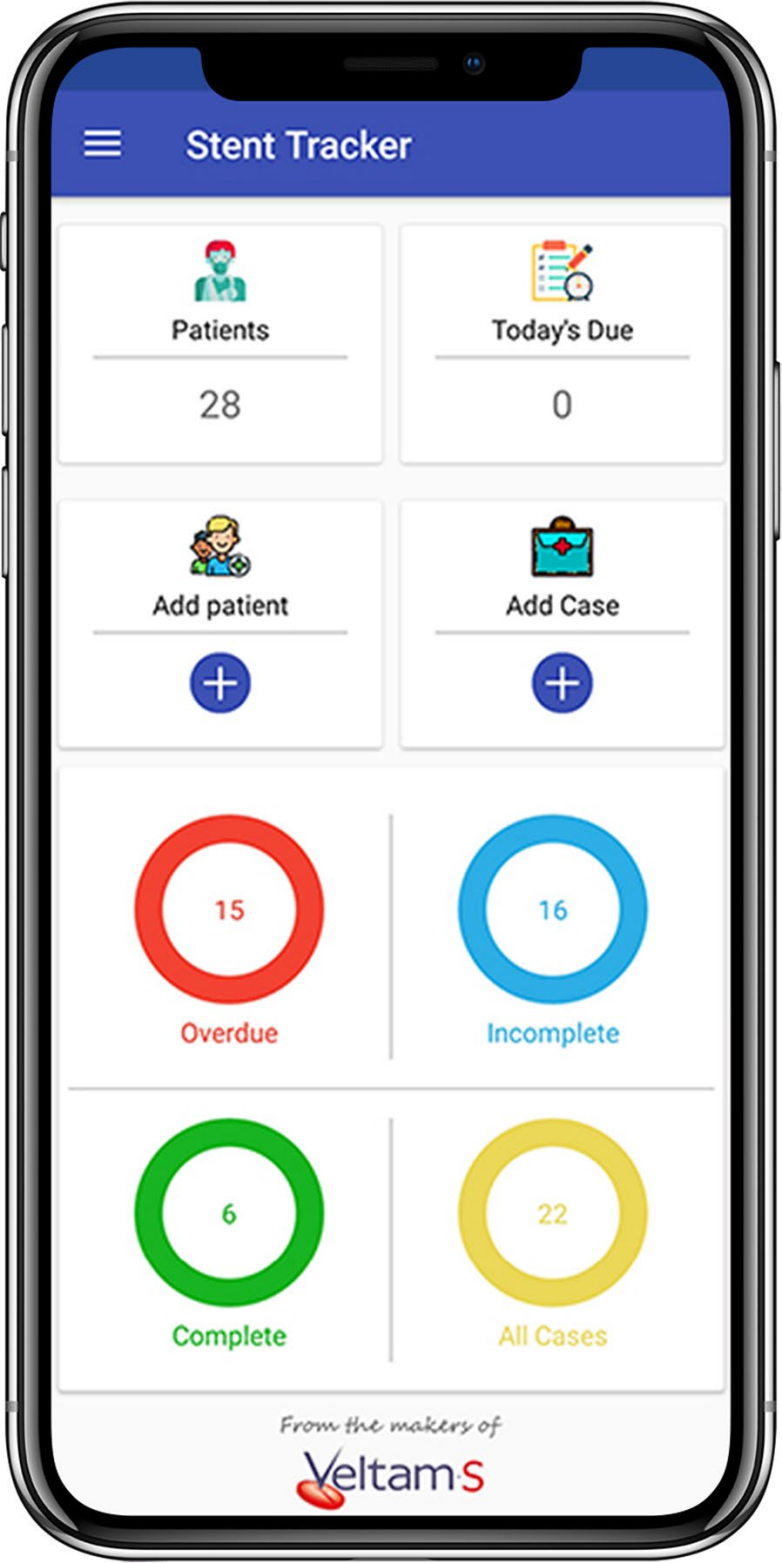
Main page of the application

**Fig. 4:**
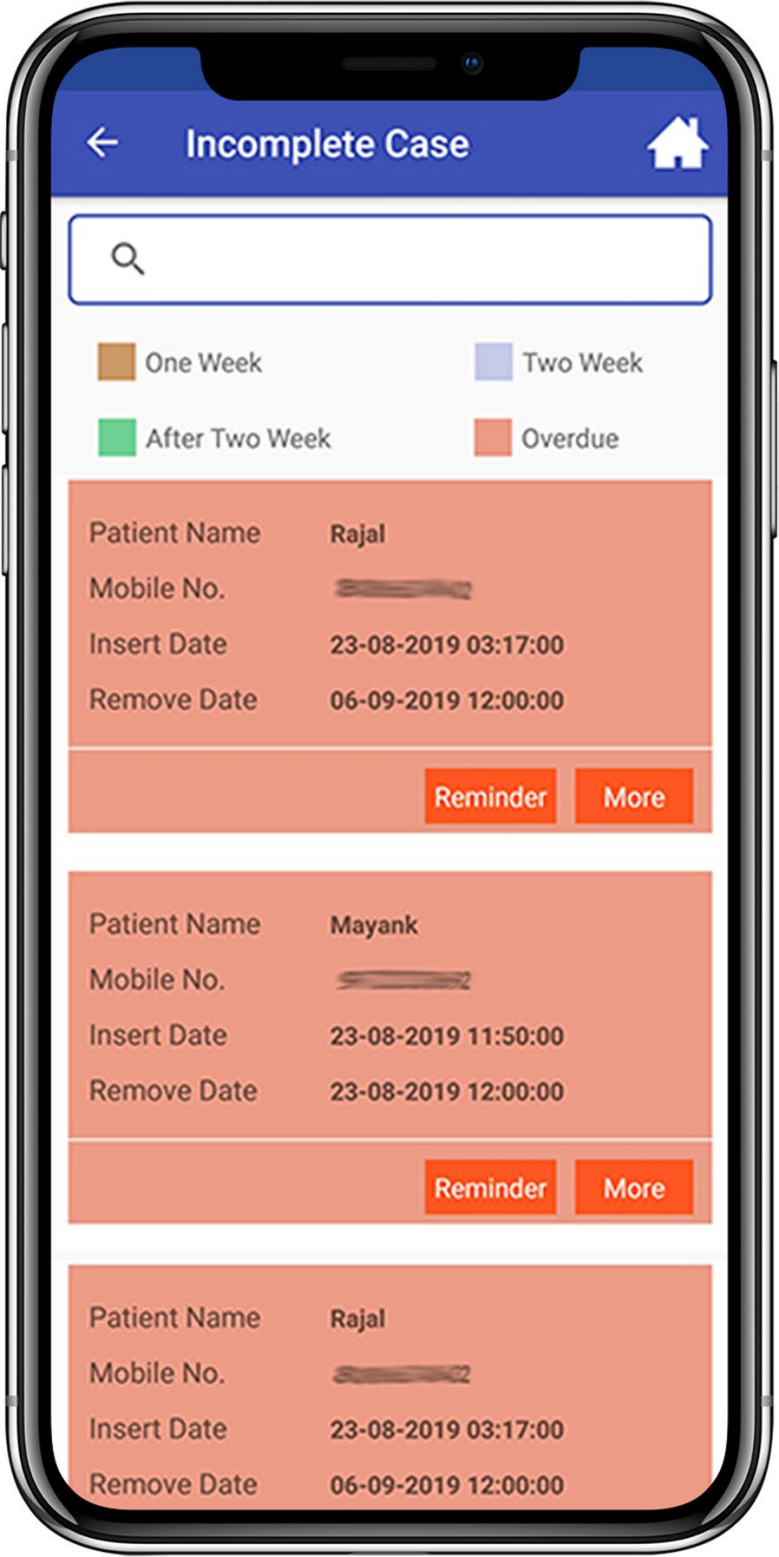
Colour-coded representation of the incomplete cases

**Fig. 5:**
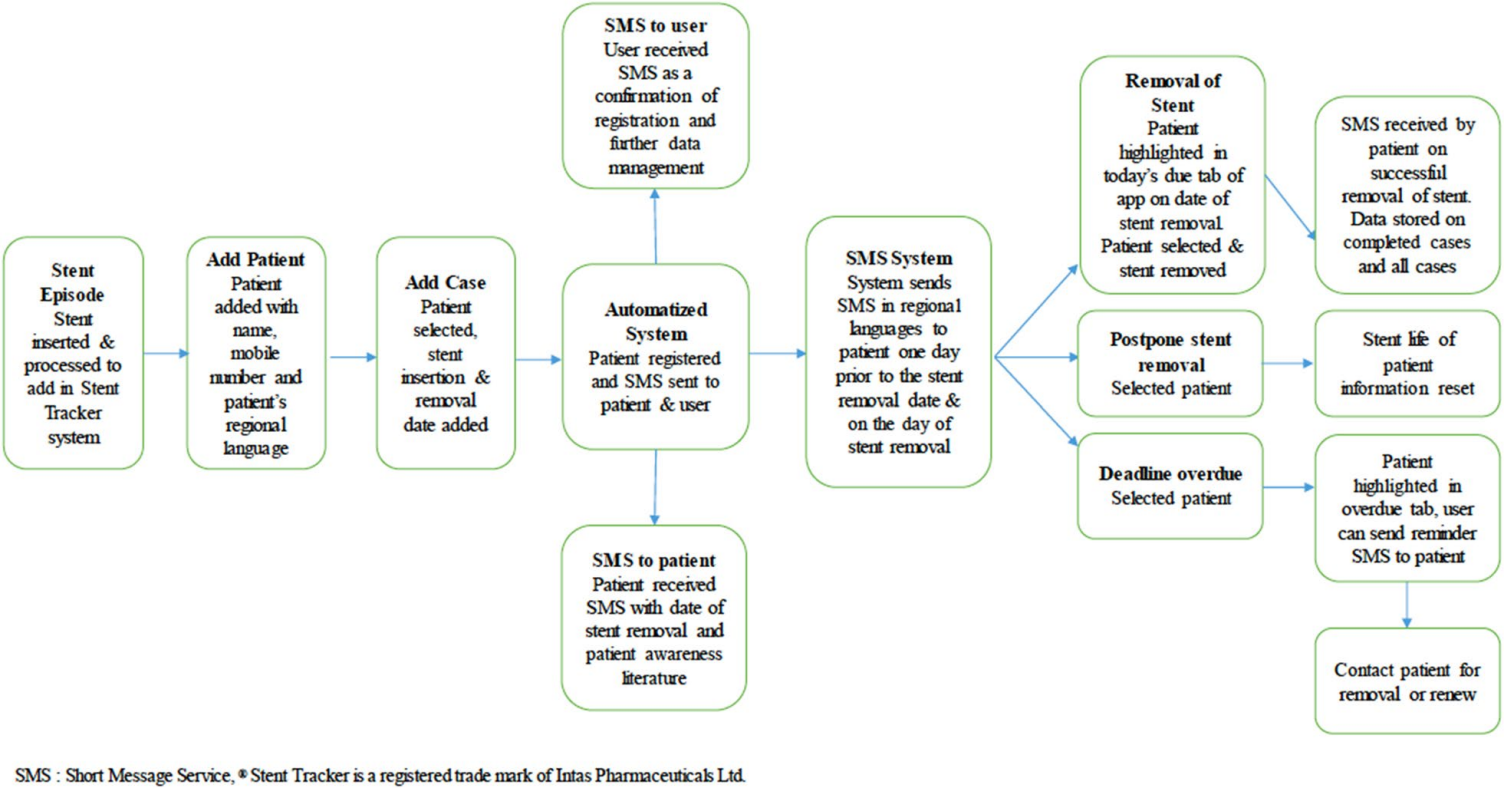
Flowchart for stent Tracker ® System

The user receives a text message (SMS) as a confirmation of registration and further data regarding management while the patient receives a SMS with date of stent removal and patient awareness literature. Stent Tracker ® system sends SMS in regional languages to patient upon registration onto the system, one day prior to the stent removal date, on the day of stent removal as well as after stent removal with name of user and hospital. The Stent Tracker ® identifies patients which have surpassed their stent removal due date and accordingly highlights them in today’s due / overdue tab of attending physician’s Stent Tracker ® system until the stent has been removed or changed and updated on the system by the user (Figs. 3, 4). User also has the option of postponement of the stent removal if required. On successful removal of stent, patient would receive a confirmatory text message. Flowchart of Stent Tracker ® system is highlighted in Fig. 5. Stent Tracker ® is available on the website as well as android/iOS platforms. Stent Tracker ® allows multiple logins; by sharing credential to nursing staff or operation theatre staff, user can make sure the timely management of overdue stents. We assessed the efficacy of the Stent Tracker ® in preventing forgotten double-J ureteral stents and further analysed the causes of overdue stents. The collected data were presented descriptively.

## Results

A total of 100 patients were stented during this period of 3 months. 71% (*n* = 71) were male, and 29% (*n* = 29) were female. Median age of the patients was 42.61 years. Three of the male patients were of paediatric age group 4.2% (*n* = 3), whose stenting was done under general anaesthesia; rest all patients were stented under local anaesthesia. Mean duration of stent in situ was 6.38 weeks. All patients received periodic messages (average of 3), regarding follow-up and date for stent removal or replacement. 20% (*n* = 20) of the patients had bilateral stenting done, 47% (*n* = 47) had only right sided stenting done, while 32% (*n* = 32) had only left sided stenting done. Most common indication for stent placement was right renal calculus 26% (*n* = 26) followed by left ureteric calculus 24% (*n* = 24), left renal calculi 23% (*n* = 23). Bilateral calculi were present in 20% of cases (*n* = 20). 3% (*n* = 3) patients were delayed for follow-up due to their personal reasons, with 2% (*n* = 2) 2 patients lost to follow-up. Of 98 patients who came for follow-up, none of the patients had any stent-related complications and were able to follow instructions provided to them via SMS text messages.

**Table:** 

Sr. No.	Indication	Number of cases
1	Bilateral	20
2	Right renal cal	26
3	Right ureteric cal	22
4	Left renal cal	23
5	Left ureteric cal	24
6	Malignancy	2
8	Pregnant females	3
9	Right pyelonephritis	7
10	Left pyelonephritis	4
11	Right pyonephrosis	4
12	Left pyonephrosis	2
13	Right ureteric stricture	2
14	Left ureteric stricture	3

**Table:** 

Renal calculi	49
Ureteric calculi	46
Pyelonephritis	11
Pyonephrosis	6
Malignancy	2

## Discussion

With the rising number of endourological procedures performed at high-volume centres such as ours, the number of patients with indwelling DJ stents has also been rising, thus increasing the probability of forgotten or retained DJ stents. Apart from the volume of patients on the part of the hospital, several other factors like loss of paper discharge cards, education level of patients, carelessness on part of the patient or miscommunications between the treating physician and the patient might contribute towards increasing the risk of retained or forgotten DJ stents. In a study conducted by Divakaruni N et al., approximately 12% of all ureteral DJ stents are retained or forgotten  [4]. Majority of the patients presenting to our department belong to the lower middle class, low economic strata, with a low education level, who either have limited access to internet or smartphones or are not skilled enough to use any complicated applications. The main advantage of the Stent Tracker application in this situation is that it sends the patient a simple text message in the regional language of the patient regarding their follow-up post-DJ stent insertion, date for DJ sent removal or replacement and literature-related patient awareness regarding DJ stents, while the treating physician or user has the access to an application which would help him track and plan the further management and reduce the risk of patient being lost to follow-up leading to a forgotten DJ stent.

In these times of the COVID-19 pandemic, the use of this application has several advantages, some of which are: it would aid the treating urologist to keep a track of the stents placed, remind both the urologist and patient regarding the need for replacement of stent if and when required and provide an integrated platform for storage of patient record which would reduce the need for a physical follow-up even if the paper discharge is lost. The patient would have a record of his next appointment in the form of a text message on his phone. There have been few studies done in the past mentioning various other methods like online registries.

In these studies done as early as 1996 by Monga et.al., [5] and by *McCahey* and Ramsden [6], they demonstrated a reduction in the number of overdue DJ stents from 3.6% to 1.1%

Ather et al. reported a significant decrease in the incidence of forgotten stents from 12.5 to 1.5% over the course of 1 year, making a strong case for the implementation of such systems into urological practice [7].

In India, Sabharwal et al. proposed a novel online registry system in 2014 [8]. But their main disadvantage was requirement of specific programmes, access to the hospital network and extra costs. Moreover, these systems are only available to their developer institutions which are usually high-volume centres [9].

The recent rapid development in the area of cellular applications has made it advantageous to use a mobile based application which would be convenient for user and can be shared with multiple institutions across widely accessible platforms. Several similar studies have been conducted in the past, such as by Mohina et al., including 194 patients and by Zeimba et al., including 115 patients in 2017, using a similar application known as Ureteral Stent Tracker (UST) developed by Visible Health Inc. (Austin, TX, USA) in partnership with Boston Scientific [9, 10]. Both studies showed promising results of only 1 and 3 patients, respectively, lost to follow-up with almost a 90% plus timely removal of stents [11]. In 2019, Volkan Ulker et al., in his study, followed up 87 patients with indwelling DJ stents. Group 1 was followed up using Ureteral Stent Tracker (UST) application while the Group 2 was followed up with normal paper discharge cards. Among patients who did not return for stent removal, statistical evaluation revealed that patients in group 1 had significantly less overdue times (*p* = 0.001) and lost to follow-up cases (*p* = 0.001) compared to group 2. [11].

A similar application known as “Urostentz” has also recently been developed by Kasturba medical college as well. [12].

## Conclusion

The use of stent tracker application proved to be advantageous over the regular OPD paper system, especially in times of COVID-19, as it significantly reduced the number of visits of the patient to the hospital, it provided a more streamlined tracking of the indwelling stents for the user, and patient compliance was found to be almost 95%. Being cloud based, it was easily accessible to the user, and with the option of sharing the account details, the patient record could be accessed by several residents from their individual devices. It added to the convenience of both the treating urologist and the patient. It has the potential to be added as an important pillar in the basic structure of record keeping of a patient in the future.

## Data Availability

It was collected from our own institute with due permission.

## Abbreviation

DJ: Double-J
